# Sex-Determining Region Y Chromosome-Related High-Mobility-Group Box 10 in Cancer: A Potential Therapeutic Target

**DOI:** 10.3389/fcell.2020.564740

**Published:** 2020-12-03

**Authors:** Liming Yu, Fan Peng, Xue Dong, Ying Chen, Dongdong Sun, Shuai Jiang, Chao Deng

**Affiliations:** ^1^Department of Cardiovascular Surgery, The First Affiliated Hospital of Xi’an Jiaotong University, Xi’an, China; ^2^Department of Cardiovascular Surgery, General Hospital of Northern Theater Command, Shenyang, China; ^3^Department of Cardiology, Xijing Hopspital, The Airforce Military Medical University, Xi’an, China; ^4^Outpatient Department of Liaoning Military Region, General Hospital of Northern Theater Command, Shenyang, China; ^5^Department of Hematology, The First Affiliated Hospital of Xi’an Jiaotong University, Xi’an, China

**Keywords:** sex-determining region Y-related high mobility group-box 10 (SOX10), cancer, developmental biology, melanoma, breast cancer

## Abstract

Sex-determining region Y-related high mobility group-box 10 (*SOX10*), a member of the *SOX* family, has recently been highlighted as an essential transcriptional factor involved in developmental biology. Recently, the functionality of SOX 10 has been increasingly revealed by researchers worldwide. It has been reported that SOX10 significantly regulates the proliferation, migration, and apoptosis of tumors and is closely associated with the progression of cancer. In this review, we first introduce the basic background of the SOX family and SOX10 and then discuss the pathophysiological roles of SOX10 in cancer. Besides, we enumerate the application of SOX10 in the pathological diagnosis and therapeutic potential of cancer. Eventually, we summarize the potential directions and perspectives of SOX10 in neoplastic theranostics. The information compiled herein may assist in additional studies and increase the potential of SOX10 as a therapeutic target for cancer.

## Introduction

Cardiovascular diseases, cerebrovascular diseases and neoplasms are three major killers among human across the world (Jiang et al., 2017, 2018a). Cancer is a primary cause of morbidity and mortality worldwide, regardless of the level of human development. An estimated 18.1 million new cancer cases and 9.6 million cancer deaths occurred in 2018 worldwide (Bray et al., 2018). The occurrence of cancer is increasing because of the growth and aging of the population, thus contributing to an enormous burden on society in both developing and developed countries. Thus far, the lack of early diagnosis tools and effective therapeutic strategies is a major contributor to the dismal survival outcome (Lieberman et al., 2016). Therefore, recent studies have focused on searching for novel therapeutic medications with a better understanding of drug targets.

Sex-determining region Y-related high mobility group-box (*SOX*) genes are highly conserved transcription factors during biological evolution (Chan et al., 2003; Castillo and Sanchez-Cespedes, 2012). In accordance with the similarity degree of the HMG structure domain, these proteins have been divided into a suite of subgroups from *SOXA* to *SOXJ*. The expression and dysfunction of SOX proteins are closely related to pathological oncogenesis. Studies have demonstrated that SOX proteins, including SOX2 (Chen et al., 2008), SOX3 (Li et al., 2013), SOX4 (Liu et al., 2006; Wu et al., 2019), SOX9 (Wang Y. et al., 2018), SOX10 (Chan, 2013), and SOX11 (Yang Z. et al., 2019), are abnormally expressed in tumors. Some SOX proteins, such as SOX2 (skin squamous-cell carcinoma) (Boumahdi et al., 2014), SOX3 (gastric cancer) (Shen et al., 2020), SOX4 (osteosarcoma) (Chen et al., 2020), SOX5 (prostate cancer) (Yang B. et al., 2019), and SOX9 (colorectal cancer) (Blache et al., 2019), are abnormally highly expressed in various kinds of tumors, whose genes play the role of oncogenes. Other SOX proteins, such as SOX1 (cervical adenocarcinoma) (Yuan et al., 2019), SOX7 (breast cancer) (Zhang et al., 2018), SOX11 (Hepatocellular carcinoma) (Liu et al., 2019), and SOX17 (Cholangiocarcinoma) (Merino-Azpitarte et al., 2017), are expressed in low levels in tumors, playing the role of tumor suppressor proteins. In the relevant progress of the SOX family member, SRY, SOX2, SOX4, and SOX9 have been extensively reviewed, and their roles in the occurrence and development of tumors have been discussed. In addition to SOX10, few relevant studies have been carried out on other molecules, which is not conducive to analysis and summary. As an essential regulator of neural crest (NC) development, SOX10 plays a vital role in the occurrence and development of tumors. *SOX10*, a member of the *SOXE* subgroup, was first discovered in mouse embryos by Wright et al. (1993). Numerous studies have suggested that SOX10 is highly expressed in melanoma (Cronin et al., 2018), breast cancer (Feng et al., 2017), bladder cancer (Yin et al., 2017), ovarian and epithelial tumors (Kwon et al., 2016), whereas its tumorigenic roles have not been clearly elucidated yet (Sun et al., 2014; Laurette et al., 2015; Kaufman et al., 2016; Seberg et al., 2017). Therefore, we have carefully summarized the recent literature to review the roles of SOX10 in cancer.

In this review, we focus on the emerging roles of SOX10 in the development of various cancers. Initially, we introduce the basic background of the SOX family and SOX10 and discuss the pathophysiological roles of SOX10 in cancer. Then, we enumerate the application of SOX10 in pathological diagnosis and therapeutic potential in cancer. Eventually, we summarize the potential directions and perspectives of SOX10 in neoplastic theranostics. Collectively, the information compiled here comprehensively characterizes the roles of SOX10 in cancer, which may potentially provide a novel theoretical basis for the early clinical diagnosis, prognosis judgment, and treatment of cancer.

## General Background of SOX10

### The SOX Family and SOX10

As a critical regulator of cell fate, the *SOX* family of transcription factors has become a powerful driver of somatic reprogramming directly to multiple developmental processes, including sex determination, muscle differentiation, hair follicle development, and blood vessel development (Bowles et al., 2000). During the long period of evolution, the *SOX* family is highly conserved in a suite of biological processes, such as cell growth, differentiation, proliferation, and apoptosis. To date, the SOX family includes *SOXB1 (SOX1-3), E (SOX8-10)*, and *F (SOX7, SOX17, SOX18)* subtypes (Guth and Wegner, 2008). The common characteristic of *SOX* is the DNA binding domain, which is similar to the Sry/HMG gene structure (Kiefer, 2007; Table 1). The *SOXE* subgroup has evolved into a unique pattern to combine targeted genes, relying on multilevel interactions with HMG, and dimerization domains. SOXE is pivotal for embryonic development, including sex differentiation (SOX8 and SOX9), glial formation (SOX8, SOX9, and SOX10), pancreatic development (SOX9 and SOX10), and NC development (SOX8, SOX9, and SOX10) (Huang et al., 2015; Lefebvre and Dvir-Ginzberg, 2017; She and Yang, 2017; Weider and Wegner, 2017; Haseeb and Lefebvre, 2019; Liang et al., 2020). Chalmel et al. (2013) found that *SOX8* and *SOX9* transcription factors are involved in sex differentiation, male gonadal development, and adult spermatogenesis maintenance. *SOX8(−/−)* mice lacking *SOX9* in the Sertoli cells were unable to form testicular cords or establish spermatogenesis. SOXE preserves basal membrane integrity to prevent testicular cord disintegration and inhibits ovarian development early in testicular development (Georg et al., 2012). Cheung and Briscoe (2003) found that SOX8, SOX9, and SOX10 could promote the formation of NC in the chick neural tube through the experiment of chick embryo misexpression. In migratory NC cells, the expression of *SOXE* gene tends to differentiate the cells into glial cells and melanocytes, and away from neuronal lineages. SOX10 is later expressed or plays a role in NC precursors in most species, so its primary role may be in later regulatory events, particularly melanocyte and glial cell formation (Haldin and LaBonne, 2010). SOX9 directly regulates Type II collagen, one of the most crucial collagen proteins in cartilage formation (McCauley, 2008). Compared with *SOX9*, the misexpression of SOX10 resulted in a massive increase in pigment cells (Aoki et al., 2003), which is consistent with the effect of the NC derivative specification. *SOX10*, located at 22q13.1, has five transcripts (splice variants), 67 orthologs, and 16 paralogs. As a nucleocytoplasmic shuttle protein, SOX10 is of great significance to the development of the neural crest, the peripheral nervous system, and melanocytes (Castillo and Sanchez-Cespedes, 2012). Recently, SOX10 has received more attention due to its involvement in the genesis and development of various cancers.

**TABLE 1:** The subgroup of SOX family.

**SOX subgroup**		**Gene**				
SOXA		SRY				
SOXB	SOXB1	SOX1	SOX2	SOX3	SOX19	
	SOXB2	SOX14	SOX21			
SOXC		SOX4	SOX11	SOX12	SOX22	SOX24
SOXD		SOX5	SOX6	SOX13	SOX23	
SOXE		SOX8	SOX9	SOX10		
SOXF		SOX7	SOX17	SOX18		
SOXG		SOX15	SOX16	SOX20		
SOXH		SOX30				
SOXI		SOX31				
SOXJ		SOXJ				

### SOX10 in Developmental Biology

The emerging characteristics of SOX10 in developmental biology have drawn much attention since the last decade (Wahlbuhl et al., 2012; Julian et al., 2017). The NC is a transient embryonic structure that gives rise to all peripheral glial cells, different peripheral neurons such as sensory and autonomic neurons, satellite glia, and Schwann cells, pigment, skeletal muscle cells, and adult stem cells (Nitzan et al., 2013). The NC cells originate from the boundary between the neural plate and the non-neuroectoderm, delaminate from the dorsal neural tube, and migrate widely throughout the body (Mayor and Theveneau, 2013). SOX10 promotes the specification of oligodendrocyte and the specification and differentiation of NC (Wegner, 2010; Weider and Wegner, 2017). SOX10 is also expressed in breast cells and exhibits a high level of stem/progenitor cell activity, including fetal and adult breast cells *in vivo* and mammary organoid cells *in vitro* (Dravis et al., 2015). SOX10 can regulate the maintenance of NC stem cells and the balance between multi-line differentiation during embryonic development (Shakhova et al., 2012). SOX10 deactivation causes the early loss of several NC derivative cell types, including melanocyte, glial cells of the peripheral nervous system and the enteric nervous system, which further results in the pathological changes of the endocrine system, peripheral nervous system, circulation system, and skeletal muscle system. Besides, *SOX10* gene mutation can lead to Kallmann syndrome and Waardenburg–Hirschsprung syndrome, which are associated with deafness and Hirschsprung disease (Pingault et al., 1998). At present, there are mainly two mutation sites found. One is close to the MITF distal enhancer; the other is located in the U1 *SOX10* enhancer (Baral et al., 2012).

SOX10 plays an essential role in the development of NC and the fate of sensory neurons *in vivo*. Delfino-Machin et al. (2017) demonstrated that SOX10 is involved in regulating the fate of dorsal root ganglion progenitor cells. Their study found a new *SOX10* mutant allele, SOX10^*baz*1^, and the number of sensory neurons was higher than the wild type, while intestinal and sympathetic neurons are almost non-existent, leading to the overproduction of sensory neurons. Data show that in zebrafish dorsal root ganglia, SOX10 plays a vital role in maintaining the balance between neurons and glial cells and plays the role of sensory neurons and glia fate specification (Delfino-Machin et al., 2017).

The role of SOX10 depends on the activation and regulation of various upstream molecules. ETS proto-oncogene 1 (Ets1), a protein encoded by the human *Ets1* gene, has versatile roles during the processes of cancer development (Kim et al., 2018). At the molecular level, Ets1 activates the SOX10-multiple-species conserved sequence 4 (MCS4) enhancer, which is critical to SOX10 expression in NC derivatives. Therefore, Ets1 may interact with SOX10 to promote proper melanocyte and enteric ganglia development (Saldana-Caboverde et al., 2015). In addition, the interaction between SOX10 and the N-myc interactor Nmi is shown in plenty of cell lineages and has been shown to regulate the transcriptional activity of *SOX10*. Nmi may be regulated by recruiting SOX10 into a cytoplasmic complex, suggesting that Nmi may be promoter specific *in vivo*. Interaction between Nmi and SOX10 can play a role in several glial cell types and their transformed derivatives (Schlierf et al., 2005). SOX10 can also act synergistically with a variety of molecules to act on the neural crest. SOX10 can regulate the production of myelin transcription and neuronal function by interacting with proteins, including oligodendrocyte transcription factor 1 (OLIG1) and early growth response protein 2 (EGR2) (Li et al., 2007; LeBlanc et al., 2007). Moreover, the interaction between SOX10 and paired box gene 3 (PAX3) is considered a regulator of genes related to symptoms of Waardenburg syndrome, particularly microphthalmia-associated transcription factor (MITF), which is a basic helix-loop-helix leucine zipper transcription factor that is involved in a lineage-specific pathway that regulates various cells, including melanocytes (Dynek et al., 2008), osteoclasts (Hershey and Fisher, 2004), and mast cells (Martina et al., 2014). MITF can facilitate NC formation. Promoter deletion and mutation analysis showed that SOX10 could promote the expression of MITF by binding to an evolutionarily conserved region between the mouse and the human MITF promoter. SOX10, in synergy with PAX3, vastly promotes the expression of MITF (Bondurand et al., 2000; Seberg et al., 2017).

**FIGURE 1:**
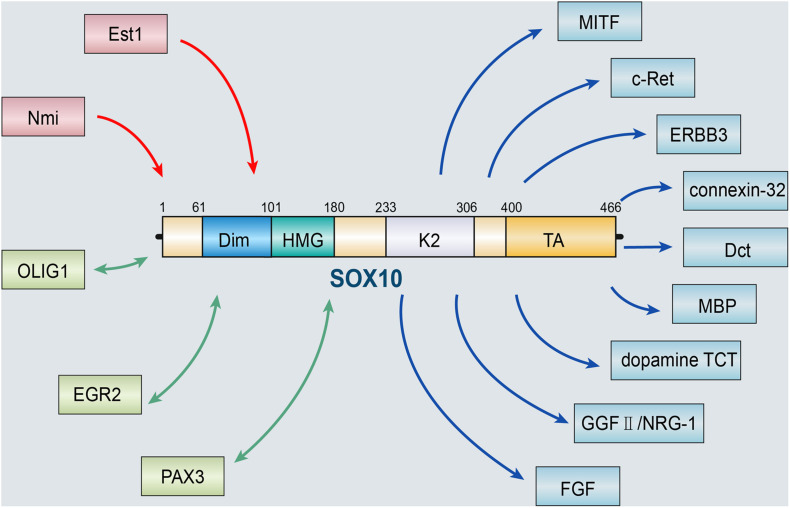
SOX10 and its upstream and downstream targets. The role of SOX10 depends on the activation and regulation of various upstream molecules, including Est1 and Nmi. SOX10 can also act synergistically with a variety of molecules, such as OLIG1, EGR2, and PAX3. Besides, several target genes of SOX10 have been identified, including MITF, ERBB3, c-Ret, and some terminal differentiation markers, such as genes for connexin-32, Dct, MBP, dopamine TCT, GGFII/NRG-1, and FGF (OLIG1, oligodendrocyte transcription factor 1; EGR2, early growth response protein 2; PAX3, paired box gene 3; MITF, microphthalmia-associated transcription factor; ERBB3, epidermal growth factor receptor 3; Dct, dopachrome tautomerase; MBP, myelin basic protein; BMP2, bone morphogenetic protein 2; GGFII, glial growth factor II; NRG-1, neuregulin-1; FGF, fibroblast growth factor).

Several target genes of SOX10 have been identified, including transcription factors MITF, receptor tyrosine kinases ERBB3 and c-Ret, and some terminal differentiation markers, such as genes for connexin-32, dopachrome tautomerase (Dct), myelin basic protein (MBP), and dopamine TCT (Britsch et al., 2001; Dutton et al., 2001; Inoue et al., 2004). Moreover, Britsch et al. (2001) confirmed that the constitutive expression of SOX10 in NCSCs retained neuronal differentiation potential. *In vivo*, SOX10 is required to maintain the expression of epidermal growth factor receptor 3 (ERBB3) in NC cells. RT-PCR analysis of NC stem cells *in vitro* showed that ERBB3 mRNA is significantly down-regulated after pre-incubation of bone morphogenetic protein 2 (BMP2) for 24 h. In contrast, this downregulation is significantly reduced in NCSCs infected with SOX10 retroviruses. The loss of gelling potential caused by BMP2 pre-incubation is related to the downregulation of glial growth factor II (GGFII)/neuregulin-1 (NRG-1) co-receptor, and overexpression of *SOX10* rescued this down-regulation to a certain extent. These data indicate that SOX10 prevented the destruction of glial potential by BMP2 by maintaining the response of NCSCs to GGFII/NRG-1 (Kim et al., 2003). Dravis et al. (2015) discovered that fibroblast growth factor (FGF) might activate SOX10 mutually. SOX10-induced FGF activation plays a significant role in the proliferation, neurogenesis, axon growth, and differentiation of neural stem cells during the development of the central nervous system. The results above all suggest that SOX10 is a pivotal regulator in developmental biology (Figure 1).

## SOX10 and Carcinogenesis

Carcinogenesis is the pathological alteration of epithelial/mesenchymal cells under the stimulation of carcinogenic factors, including inflammation, chemicals, and radiation (Vineis et al., 2010; Grivas et al., 2015; Skvortsova, 2015). Tumors are affected by numerous external factors through the metabolism, activation, and production of active carcinogenic substances (Kastan, 2008; Yang et al., 2017; Jiang et al., 2018b). In the 1970s, Sell et al. (2016) proposed that tumors are a developmental biological problem. Tumors originate from normal stem cells of the body, and the occurrence of tumors is a disorder of differentiation, not reverse differentiation, namely, abnormal differentiation (Reya et al., 2001; Jiang et al., 2018c). SOX10 may have a certain promoting effect on tumor progression. Kaufman et al. (2016) investigated the zebrafish melanoma model and found the NC progenitor (NCP) transcription factors, including SOX10, can regulate the expression of crestin. Notably, NCP gene activation can lead to the occurrence of melanoma. Compared with the control group, overexpress of SOX10 in melanocytes significantly accelerated the incidence of melanoma, emphasizing the promoting effect of SOX10 in melanoma formation (Kaufman et al., 2016).

Moreover, SOX10 plays an essential role in the proliferation, migration, and apoptosis of tumors. Yin et al. (2017) analyzed the effect of SOX10 expression on cancer cell growth by studying 31 bladder cancer samples and found that the proportion of upregulated SOX10 protein expression in bladder cancer tissues was 74.4%. The high SOX10 expression was related to the clinical stage (*P* = 0.008), T stage (*P* = 0.004), histological grade (*P* = 0.002), and lymph node metastasis (*P* = 0.006). Besides, *SOX10* knockdown in bladder cancer cells can inhibit proliferation, migration, and invasion, and SOX10 may promote bladder cancer progression by altering the expression of catenin and Met (Yin et al., 2017). Furthermore, the level of SOX10 is not only related to the degree of tumor progression but can also determine the stage of tumor.

## Roles of SOX10 in Diverse Human Neoplasms

### Melanoma

Melanoma, which develops from pigment-containing cells known as melanocytes, is the deadliest form of skin cancer and strikes tens of thousands of people each year worldwide (Owens, 2014; Moon et al., 2017). Melanoma episodes mainly occur in white populations, whereas pigmented populations from Afro-Asia have low incidence rates. Data from the National Cancer Institute concludes that melanoma is steadily increasing among whites, with a 60% increase observed within the last 30 years. The mortality of melanoma is exceptionally high, especially metastatic melanoma, with a mean survival of only approximately 6 months and a 5 years survival rate of < 5% (Gray-Schopfer et al., 2007). Malignant melanoma was once one of the most challenging tumors to treat, with few treatment options, including chemotherapy, radiation, and early immunotherapy (Houghton and Polsky, 2002). Over the past decade, researchers have found that mutations in several genes are linked to melanoma development. Researchers have found that nearly half of all melanoma patients have a mutated BRAF gene (Kefford et al., 2002; Pollock et al., 2003). Thus, the natural mutation of the BRAF gene is a hotspot target for scientists to study resistance to melanoma, and the inhibitor of mutated BRAF enzyme Vemurafenib and Dabrafenib have been marketed successively (Jordan and Kelly, 2012; Patel et al., 2020). Later, FDA approved Trametinib, which is different from the first two in that it is a MEK inhibitor, and the MEK and mutant BRAF share a partially overlapping signaling pathway, the MEK inhibitors can also be used in the treatment of BRAF mutation of melanoma patients (Leonowens et al., 2014). Combination therapy is a developing trend in tumor therapy because the pathogenesis of tumors is complex. However, it is difficult for a single drug to affect multiple targets. In 2014, FDA approved the combination of Trametinib and Dabrafenib, which can block different sites on the same signaling pathway. Clinical studies have shown that this combination therapy for treating melanoma is twice as effective as a single drug (Homet Moreno et al., 2016). Although various chemotherapy drugs have displayed certain antitumor properties, the efficacy is not pleasing. Therefore, a novel drug target is needed to elucidate this problem.

SOX10 can promote the synthesis of melanin and the proliferation of melanocytes. In melanocytes, the most typical transcription target of SOX10 is MITF. Numerous studies have demonstrated that SOX10 can directly activate MITF transcription in NC cells (Harris et al., 2010). SOX10 is an essential component of the MITF binding regulatory element in melanocytes. Seberg et al. (2017) confirmed that SOX10 could upregulate the expression of MITF by binding to PAX3. Then, SOX10 and MITF can interact with each other to activate dopachrome tautomerase and tyrosinase and further promote melanogenesis (Rooper et al., 2016; Seberg et al., 2017). SOX10 also promotes the proliferation of skin melanocytes by activating the DNA replication licencing factor minichromosome maintenance protein 5 (MCM5). In melanocytes, the deletion of SOX10 inhibits cell proliferation and the expression of MCM5 (Su et al., 2017). *In vitro* analysis showed that SOX10 could control gene transcription required for melanin synthesis, including dopachrome tautomerase (Potterf et al., 2001; Jiao et al., 2004), tyrosinase (Hou et al., 2006), and Tyr-related protein 1 (Tyrp1) (Murisier et al., 2006).

Recently, it has been suggested that SOX10 is highly relevant to melanoma development (Huang et al., 2015). Blokzijl et al. (2016) recruited 110 melanoma patients and discovered that the SOX10 assay’s specificity in serum was high. Similarly, elevated serum SOX10 is found at a high frequency among melanoma patients. In patients with metastatic melanoma, the lack of SOX10 detection is associated with better therapeutic outcomes. A change from SOX10 positivity to undetectable levels was observed in two responding patients before the response was evident clinically (Blokzijl et al., 2016). Moreover, the model system showed that melanoma cells exhibit a NC gene expression signature, which includes SOX10 expression, and that a super-enhancer is located at the *SOX10* locus in human melanoma (Kaufman et al., 2016). Moreover, Gambichler et al. (2016) discovered that the expression of SOX10 is significantly increased in primary melanoma, lymph node metastasis, and distant metastasis compared with that in melanin cell nevus, supporting SOX10 as a potent positive predictor of melanoma.

SOX10 has a principal impact on the progression of melanoma, involving the survival, migration, proliferation, invasion, and metastasis of melanoma cells (Seong et al., 2012; Lv et al., 2015; Table 2). SOX10 binds and interacts with MITF or other pro-differentiation transcription factors (Sommer, 2005; Graf et al., 2014), thereby promoting the development of melanoma. Besides, Kaufman et al. (2016) examined multiple normal and malignant cell types and found that the enrichment of H3K27Ac signaling at SOX10 is specific to melanoma cells and human ES-derived NC cells (hNCCs). Additionally, they discovered that the overexpression of SOX10 in melanocytes accelerates melanoma formation by activating NC progenitor genes and super enhancers (Murisier et al., 2006; Kaufman et al., 2016). Moreover, Sun et al. (2014) demonstrated that SOX10 can inhibit the activation of transforming growth factor (TGF) scaffold signaling in melanoma cells and promote resistance to BRAF and mitogen-activated protein kinase (MAPK) and extracellular signal-regulated kinase (ERK) phosphorylating kinase (MEK) inhibitors, contributing to the drug resistance against the acute cytotoxic effect of Raf inhibitors (Sun et al., 2014; Han et al., 2018). In addition, expression of SOX10 and melanoma inhibitory activity (MIA) are tightly correlated in melanoma cell lines. *SOX10* knockout downregulates the expression, activity, and migration of melanoma cells (Graf et al., 2014). Additionally, Shakhova et al. (2012) reported that the inhibition of *SOX10* inhibits the formation and malignant transformation of congenital melanocytic naevi as well as the expansion of melanoma, suggesting that *SOX10* inhibition may represent a molecular target for neoplasms. Moreover, Lv et al. (2015) detected the endogenous interaction between the F-box and beta-transducin repeat domain-containing 7α (Fbxw7α) ligases and SOX10 in melanoma cells. Fbxw7α promotes ubiquitination mediated SOX10 turnover through the cyclobutane pyrimidine dimer domain of SOX10, thereby inhibiting tumor progression. However, reduced Fbxw7α might contribute to the upregulation of SOX10 in melanoma cells. These authors discovered that SOX10, as the substrate of Fbxw7α-binding E3 ubiquitin ligase, could promote the migration of melanoma cells mediated by Fbxw7α (Lv et al., 2015).

**TABLE 2:** Roles of SOX10 expression in different tumors.

**Cancer type**	**Positive cases/all cases**	**SOX10 expression**	**Outcome**	**References**
Melanoma	–	↑	SOX10 promotes the survival, proliferation, and metastasis of melanoma cells	Cronin et al., 2013; Shakhova et al., 2015; Tacha et al., 2015; Han et al., 2018; Coe et al., 2019
Breast cancer	–	↑	SOX10 promotes breast cancer progression, whereas *SOX10* knockout may provide beneficial effects for treating breast cancer	Cimino-Mathews et al., 2013; Ivanov et al., 2013; Pomp et al., 2015; Harbhajanka et al., 2018; Tozbikian and Zynger, 2019
Bladder cancer	–	↑	*SOX10* knockdown significantly impacts proliferation, migration, and invasion in bladder cancer cells. SOX10 promotes bladder cancer progression by altering β-catenin and Met expression	Wiese et al., 2017; Yin et al., 2017
Hepatocellular carcinoma	–	↑	SOX10 promotes the combination of TCF4 and β-catenin to form a stable SOX10/TCF4/β-catenin complex and transactivate its downstream target genes, thereby promoting the proliferation of hepatocellular carcinoma cells	Zhou et al., 2014; Kato et al., 2017; Peng et al., 2019; Xie et al., 2019
Schwannoma	–	↑	SOX10 has a broad prospect in the diagnosis and prognosis of soft tissue tumors as a new immunohistochemical marker	Roh et al., 2006; Hirose et al., 2012; Karamchandani et al., 2012; Doddrell et al., 2013; Miettinen et al., 2015
Ovarian epithelial tumors	–	↑	Nuclear SOX10 expression is an independent indicator of poor prognosis in ovarian adenocarcinomas, especially in high-grade serous adenocarcinomas	Mohamed et al., 2013; Kwon et al., 2016
Primary adnexal tumors of the skin	–	↑	SOX10 is highly expressed in secretory cells and muscle epithelial cells of the exocrine gland	Lezcano et al., 2017; Bush et al., 2019
Pleomorphic adenoma	10/100	–	SOX10 is a novel marker for diagnosing and understanding the histogenesis of salivary gland tumors	Ohtomo et al., 2013
Acinic cell carcinoma	8/13	–	The use of SOX10 may increase the diagnostic accuracy of oncocytic lesions in acinic cell carcinoma	Schmitt et al., 2015
Sweat ductal/glandular neoplasms	9/10	–	SOX10 immunohistochemistry may distinguish some of the varying adnexal tumors from each other, and from basal cell carcinoma	Cassarino et al., 2017

Apoptosis is also referred to as programmed cell death controlled by multiple apoptosis-associated genes (Nagata, 2018). Anti-apoptosis is one of the most prominent features of tumors and is highly related to SOX10 in tumor biology. siRNA silencing of *SOX10* may contribute to the G1 phase arrest of melanoma cells via inhibiting the expression of cyclin/cyclin-dependent kinases (CDK) complex, suggesting the antineoplastic effects of *SOX10* knockdown (Shakhova et al., 2012). Another study showed a significant increase in the number of apoptotic cells treated with *SOX10* short hairpin RNA (shRNA). In addition, the apoptosis of activated caspase-3-positive cells is increased. Notably, You et al. (2017) found that astragal can induce the apoptosis of A375P and sk-mel-2 melanoma cells via inhibiting SOX10 signaling. In melanoma cells, stable *SOX10* knockout contributes to G1 phase arrest and the reduced expression of MITF and p21 but elevates p27. Stable *SOX10* knockout reduces the level of E2F transcription factor 1 (E2F1) in melanoma cells (Cronin et al., 2013). In summary, SOX10 plays a considerable role in the survival, proliferation, metastasis, apoptosis of melanoma cells, and melanoma diagnosis. The inhibition of SOX10 may be a potential effective way to treat melanoma.

### Breast Cancer

Breast cancer is a heterogeneous disease comprising multiple tumor entities associated with distinctive histological patterns, different biological features, and clinical behaviors (Weigelt and Reis-Filho, 2009). Worldwide, breast cancer affects approximately 12% of women and is the most common invasive cancer among women (Nelson et al., 2009; McGuire et al., 2015; Disease et al., 2016). As a transcription factor, *SOX10* is mainly expressed in triple-negative and metaplasia breast cancer. It can regulate the status of stem/progenitor cells and mesenchymal cells in breast tissue, and its expression in triple-negative breast cancer (TNBC) tissues is significantly higher than in other breast cancer subtypes (Cimino-Mathews et al., 2013; Nelson et al., 2017; Table 2).

SOX10 may play a regulatory role in mammary epithelial cells. *SOX10* knockout lowers stem/progenitor activity, whereas the ectopic activation of *SOX10* can induce the process of epithelial-mesenchymal transition (EMT), which means the reversible developmental transdifferentiation of polarized epithelial cells to mesenchymal cells (Dravis et al., 2015; Diaz and de Herreros, 2016; Tulchinsky et al., 2018). SOX10 increases the activity of stem/progenitor cells in TNBC cells by inducing nestin expression. *SOX10* knockout reduces the rate of CD24^–^/CD44^+^ cells and cancer stem cell properties, which ultimately inhibits tumor formation (Feng et al., 2017). Panaccione et al. (2016, 2017) identified a highly conserved *SOX10*/prominin-1 (CD133) gene marker in the clinical breast cancer data set. Their results implied that basal breast cancer progression is stimulated by *SOX10*^+^/CD133^+^ cells, which express neural stem cell-specific markers and have characteristics of cancer stem cells derived from the neural crest. Their results also provided clinical information for the development of cancer stem cell-targeted therapy (Ivanov et al., 2013; Panaccione et al., 2016, 2017). The expression of nestin mRNA is positively correlated with the expression of *SOX10* mRNA in breast cancer. SOX10 enhances the expression of nestin by directly combining with the promoter of nestin and upregulates the cancer stem cell characteristics of TNBC cells (Feng et al., 2017). In addition, *SOX10* overexpression can lead to the mesenchymal transformation of breast cells (Dravis et al., 2015). These results indicate that *SOX10* knockout/knockdown may provide beneficial effects for treating breast cancer.

### Other Neoplasms

**FIGURE 2:**
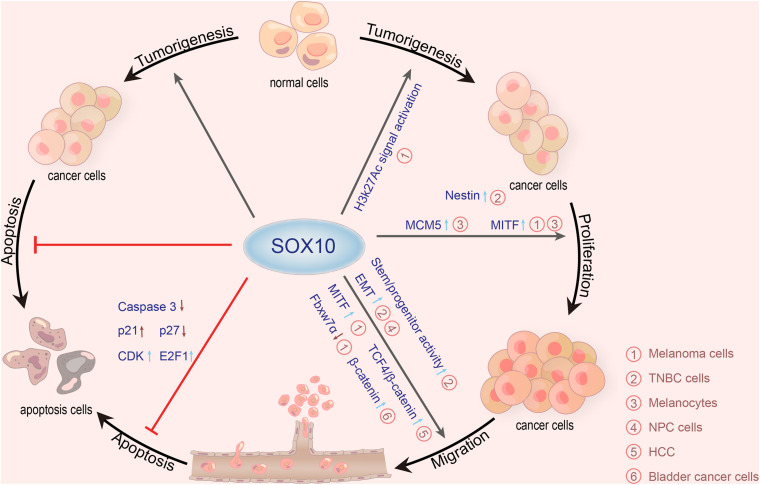
Roles of SOX10 in various cancers. SOX10 exerts carcinogenetic actions via promoting tumorigenesis, proliferation, migration, and anti-apoptosis mechanisms. SOX10 plays tumorigenic roles in melanoma cells by stimulating H3K27Ac signaling. SOX10 also enhances the expression of nestin by directly combining with the promoter of nestin and upregulates the cancer stem cell characteristics of TNBC cells. In melanocytes, the deletion of SOX10 inhibits the expression of MCM5 to inhibit cell proliferation. Besides, numerous studies have demonstrated that SOX10 can directly activate MITF transcription to make melanocyte proliferation and melanoma migration, thereby promoting the development of melanoma. *SOX10* knockout also inhibits the migration of TNBC cells and NPC cells by stimulating the EMT process. SOX10 increases the activity of stem/progenitor cells in TNBC cells by inducing nestin expression. Moreover, SOX10 promotes the combination of TCF4 and β-catenin to form a stable SOX10/TCF4/β-catenin complex, promoting the proliferation of HCC. SOX10 also can promote the migration of melanoma cells mediated by Fbxw7α. *SOX10* knockdown in bladder cancer cells can inhibit migration and invasion of bladder cancer by upregulating the expression of β-catenin. SOX10 can inhibit the apoptosis of melanoma cells by reducing the expression of caspase-3 and p27 but elevates p21, CDK, and E2F1 (SOX10, sex-determining region Y-related high mobility group box 10; MITF, microphthalmia-associated transcription factor; MCM5, minichromosome maintenance protein 5; Fbxw7α, F-box and beta-transducin repeat domain-containing 7α; CDK, cyclin-dependent kinases; E2F1, E2F transcription factor 1; EMT, epithelial-mesenchymal transition; HCC, hepatocellular carcinoma; TCF4, transcription factor 4; TNBC, triple-negative breast cancer; NPC, nasopharyngeal carcinoma).

Additionally, SOX10 is also involved in the growth and proliferation of other neoplasms, such as adenoid cystic carcinoma (Ivanov et al., 2013), mammary analog secretory carcinoma (Hsieh et al., 2016), sweat ductal/glandular neoplasms (Cassarino et al., 2017), low-grade salivary duct carcinoma (Hsieh et al., 2016), sialoblastoma (Hsieh et al., 2016), basal cell adenocarcinoma (Kato et al., 2017), basal cell adenoma (Hsieh et al., 2016), and pleomorphic adenoma (Ohtomo et al., 2013). Moreover, SOX10 is highly expressed in secretory cells and myoepithelial cells of the exocrine gland (Lezcano et al., 2017; Li et al., 2018). One study, including 174 tissue microarray cases, showed that the expression of SOX10 is increased in most schwannomas and neurofibromas (Karamchandani et al., 2012). In addition, the expression of SOX10 in ovarian epithelial tumors corresponds to the low overall survival rate. The expression of SOX10 in ovarian adenocarcinoma is significantly higher than that in benign and borderline adenocarcinoma. Kwon et al. (2016) discovered that SOX10 is expressed in most ovarian cancers and has different expression patterns in different histological types. Both serous and clear cell types have nuclear and cytoplasmic staining, while mucinous and endometrioid types only have cytoplasmic staining. Mohamed et al. (2013) found that nuclear SOX10 expression is positively correlated with chemotherapeutic resistance, and nuclear SOX10 staining is also significantly correlated with shorter overall survival, which is an independent marker of poor prognosis for serous adenocarcinoma and all ovarian adenocarcinomas. The Wnt/β-catenin signaling pathway is critical for embryonic development and normal tissue homeostasis. Similarly, increasing evidence has indicated that the abnormal activation of Wnt/β-catenin signaling is involved in carcinogenesis (Tammela et al., 2017; Wang B. et al., 2018). Zhou et al. (2014) reported that SOX10 is remarkably upregulated in hepatocellular carcinoma (HCC). SOX10 promotes the combination of transcription factor 4 (TCF4) and β-catenin to form a stable SOX10/TCF4/β-catenin complex and transactivate downstream target genes, promoting the proliferation of HCC. Additionally, SOX10 is overexpressed in bladder cancer and correlated with the malignant behavior of bladder cancer cells. Yin et al. (2017) found that *SOX10* knockout may stimulate the proliferation of bladder cancer. For further proof, these authors prepared a *SOX10* knockdown model in T24 and 5,637 bladder cancer cells by using the small interfering RNAs (siRNAs) method. Compared with the control groups, the proliferation of T24 and 5,637 cells in *SOX10*-silencing groups is significantly inhibited. He and Jin (2018) detected SOX10 expression in nasopharyngeal carcinoma (NPC) tissue by quantitative real-time PCR (qRT-PCR) and western blot, detected the invasiveness of NPC cells with knockdown of *SOX10* by MTT, flow cytometry, and transwell migration and invasion assays, and performed nude mouse tumorigenicity experiments. They then confirmed that SOX10 contributed to the progression of NPC. Experiments by Tang et al. (2018) revealed that after the knockdown of *SOX10* by siRNA in prostate cancer cells, the proliferation of prostate cancer cell lines, PC3 and DU145, is significantly inhibited, showing that SOX10 may promote the progression of prostate cancer by accelerating the proliferation and invasion of prostate cancer cells. SOX10 levels are significantly high in NPC tissues and a series of nasal pharyngeal cancer cell lines. In *in vitro* experiments, *SOX10* knockout observably inhibits the proliferation, migration, and invasion of NPC cells, as well as the EMT process. *In vivo* experiments further confirmed that *SOX10* knockout inhibits the growth and metastasis of NPC (He and Jin, 2018). To sum up, most literature has demonstrated that SOX10 is a useful immunohistochemical marker in tumorigenesis and participates in the survival, proliferation, and metastasis of diverse tumors (Table 2 and Figure 2).

## Clinical Application and Future Perspectives

### Pathological Diagnosis

Accumulating evidence has demonstrated that SOX10 is involved in the generation, survival, and maintenance of NC cells. Due to the limited expression of SOX10, it has proven to be a useful immunohistochemical marker with extensive diagnostic applications in surgical pathology (Ordonez, 2013). SOX10 is expressed at low levels in humans’ benign tissues, including melanocytes, breast tissue, cranial ganglion, dorsal root ganglion, and ear vesicle. It is also a key marker in melanoma (Bondurand et al., 1998), breast cancer (Cimino-Mathews et al., 2013), E glioma (Nonaka et al., 2008), and benign tumors such as schwannomas (Karamchandani et al., 2012). Notably, expression of SOX10 varies mainly in different stages of solid tumors, indicating that SOX10 expression disorder is involved in the occurrence and development of multiple tumors (Mohamed et al., 2013). Additionally, one study has suggested that SOX10 is an extremely sensitive diagnostic marker for primary and metastatic melanoma, with sensitivity from 97 to 100% (Ordonez, 2014). The expression of SOX10 increases with tumor progression, including mesenchymal melanoma, tumors with Schwann cell differentiation, and salivary gland tumors, especially those with myoepithelial differentiation (Ordonez, 2013). Another study revealed that the positive rates of SOX10 in monomorphic adenoma, adenoid cystic carcinoma, adenoid cell carcinoma, polymorphous low-grade adenocarcinoma, and polymorphous adenoma are 100, 96, 96, 88, and 79%, respectively, suggesting that SOX10 can be used to assist in the diagnosis of salivary gland tumor subtypes (Ohtomo et al., 2013). Moreover, Kwon et al. (2016) confirmed that SOX10 expression is an independent indicator of poor prognosis in ovarian adenocarcinoma, especially in advanced serous adenocarcinoma. Similarly, SOX10 is also recognized as a marker for diagnosing primary breast cancers, especially those with basal-like, triple-negative phenotypes. In addition, SOX10 can improve the diagnostic accuracy of fine-needle aspiration tumor lesions (Schmitt et al., 2015). By comparing human melanoma black 45 (HMB45), Melan A, and S-100 and SOX10 as a marker of melanoma in effusion cytology, Dermawan et al. (2019) confirmed that SOX10 has a strong specificity and sensitivity for melanoma, and the combination of HMB45 and SOX10 can be used as the preferred staining agent for the diagnosis of exuded melanoma in cytology. Therefore, SOX10, as a new immunohistochemical marker, has broad prospects in the diagnosis and prognosis of tumors.

### Theranostics

Theranostic is an emerging field of medicine that combines treatment and diagnosis to personalize the treatment and diagnosis for patients simultaneously or sequentially. As a novel marker, SOX10 has recently been revealed by many researchers to be uniquely advantageous to the direction of treatment. Mutant melanoma cells may prompt an adaptive resistance, which rapidly triggers the survival signals to protect against the cytotoxic effects of RAF inhibitors until acquired resistance takes over. FOXD3 is a critical mediator of adaptive resistance in mutant BRAF melanoma cells. FOXD3 depletion enhances the cytotoxic effect of RAF inhibitors in mutant BRAF melanoma cells (Basile et al., 2012). Han et al. (2018) found that knockdown of *SOX10*, the upstream regulator of FOXD3, can sensitize mutant BRAF melanoma cells to Vemurafenib *in vitro* and *in vivo*, suggesting that SOX10 can protect melanoma cells against the acute cytotoxic effect of RAF inhibitors. Notably, their work focused on the immediate (within 120 h) effects of SOX10 depletion on the survival of Vemurafenib-challenged cells, which is more pertinent to the time window of adaptive resistance (Han et al., 2018), revealing that the expression of SOX10 help to predict the development of resistance to BRAF inhibitor therapy. Patients with TNBC are at increased risk for visceral metastases and other primary non-breast cancers, especially lung cancer. Due to the lack of TNBC standard immunoassay, it may be challenging to identify TNBC metastatic and primary cancers from other organs. SOX10 participates in the maintenance and differentiation of stem cells through the Notch signaling pathway and regulates the characteristics of tumor stem cells in TNBC cells by inducing the expression of nestin at the mRNA and protein levels (Feng et al., 2017). Therefore, it may be possible to use this feature to identify the origin of the tumor. Laurent et al. (2019) found that SOX10 can be stably expressed in breast cancer metastasis sites, especially lung cancer metastasis sites. Considering its uniqueness, SOX10 seems to be a good marker to distinguish TNBC from lung adenocarcinomas, especially for patients with a history of breast cancer, which can establish the mammary gland source of tumor nodules in the organs from endoderm and mesoderm. This method should be considered for diagnostic examination of pulmonary nodules, thus guiding the next treatment and avoiding a poor prognosis of TNBC under certain conditions (Laurent et al., 2019). Merelo Alcocer et al. (2019) retrospectively analyzed 20 cases of benign lymph nodes and 20 cases of granulomatous dermatosis and performed SOX10 immunohistochemical assay on them. The results showed that SOX10 positive cells were detected in granulomatous dermatosis and benign lymph nodes. Moreover, SOX10 positive cells in lymph nodes were mainly distributed in the subcapsular and medullary sinus. Therefore, SOX10 is a good stain for evaluating metastatic melanoma. Notably, since SOX10 staining is strongly positive in granulomatous dermatosis, it may confuse the diagnosis when evaluating melanoma reresection and micrometastasis, so additional evidence is needed to supplement and support the diagnosis (Merelo Alcocer et al., 2019). Moreover, perineural invasion (PNI) is associated with a high risk of keratinocyte carcinoma. In Mohs surgery, the determination of PNI for staging and post-adjuvant therapy is important and difficult. To confirm or exclude suspected PNI in hematoxylin and eosin sections, Donaldson and Weber performed double immunohistochemical staining on Mohs frozen sections, using the neural marker SOX10 in combination with the pan-cellular keratin marker AE1/AE3. Among the 23 cases of Mohs suspected of PNI, 18 cases were confirmed as definite nerve involvement by double staining, which proved that double staining of frozen tissue was feasible and could be used for real-time detection of PNI during Mohs, demonstrating the important role of SOX10 in diagnosis and therapy (Donaldson and Weber, 2019). Today, more and more assistive tests are entering clinical practice, so the demand for cell blocks will continue to grow. If additional biopsies are required, the cost will be significantly increased, and treatment will be delayed. Besides, there may be a significant risk to patients due to different anatomical locations. Therefore, it is of great significance for a clinical diagnosis to obtain a definite diagnosis by preserving the limited material of cell blocks. Mito et al. (2018) conducted immunohistochemistry (IHC) tests on 34 melanomas, 31 epithelioid/pleomorphic sarcomas, and 42 cancer specimens. IHC performed SOX10 with peroxidase-based brown chromogen and AE1/AE3 with alkaline phosphatase based red chromogen for all specimens. The results showed that SOX10/keratin bicolor IHC is an effective, sensitive, and specific method to identify a melanoma, sarcoma, and carcinoma. This approach can identify melanomas and reduce the number of markers needed to detect epithelioid malignancy, potentially reducing costs, and preserving valuable tissue to guide adjunctive therapeutic research (Mito et al., 2018). In summary, SOX10 may become a potential theranostic target for cancer.

### Therapeutic Targets

Given the ability of SOX10 to be involved in tumorigenesis and tumor progression, it is evident that SOX10 can be considered a desirable therapeutic target for multiple types of cancer. The investigations into the mechanisms of SOX10 may lead to the development of novel therapeutic targets for treating and preventing human cancer. SOX10 plays a pivotal role in the development of NC in vertebrates and is involved in the establishment and maintenance of melanocyte lineage. SOX10 can inhibit the activation of TGF in melanoma cells and promotes resistance to BRAF and MEK inhibitors (Nazarian et al., 2010; Hodis et al., 2012; Wagle et al., 2014; Fallahi-Sichani et al., 2017). Cronin et al. (2018) found that *SOX10* knockout activates TGF-β signaling and promotes resistance to BRAF and MEK inhibitors in melanoma cells. Furthermore, cancer progression may employ altered phosphorylation of pivotal transcription factors, and posttranslational phosphorylation is one mechanism that governs transcription factor activity, implying that posttranscriptional control regulation of *SOX10* is likely to develop an effective treatment for melanoma (Cronin et al., 2018). In addition, *SOX10* knockout increases the resistance of melanoma to antineoplastic drugs by increasing the expression of receptor tyrosine kinase epidermal growth factor receptor (EGFR) (Shakhova, 2014; Shaffer et al., 2017). Seong et al. (2012) discovered a cadre of downstream SOX10 signaling molecules, such as MITF or melanocortin 1 receptor in B16 melanoma cells, involved in the migration and metastasis of melanoma cells, providing a potential target for therapeutic intervention. At present, the application of SOX10 mainly focuses on disease diagnosis and prognosis assessment. There are no proven effective inhibitors or antibodies in the targeted therapy of clinics in the context of cancers. Taken together, these studies indicate that the clinical application of SOX10 is promising to limit the genesis and progression of various human cancers in the future as our knowledge of SOX10 targeting approaches continuously develops.

### Future Perspectives

Although scientists have been studying SOX10 for many years and have discovered many functions of SOX10 and its downstream signaling pathways, it remains elusive for further research. Further investigations of SOX10 may focus on (i) whether the abnormal expression of SOX10 carries the risk of stimulating tumorigenesis; (ii) whether alterations in SOX10 levels can identify grade malignancy; (iii) how to specifically silence *SOX10* in tumors; (iv) the potential adverse effects of *SOX10* knockout; (v) how to maintain SOX10 at appropriate levels to modulate oncogenesis. Herein, further investigations are required before SOX10 can be used clinically as essential diagnostic targets, and SOX10-targeted drugs can be considered clinically valuable therapeutic targets in clinical oncology.

## Conclusion

Overall, SOX10 has been recognized as a useful immunohistochemical marker with extensive diagnostic applications in surgical pathology (Ordonez, 2013). SOX10 is involved in the formation of neural crest, peripheral nervous system (Castillo and Sanchez-Cespedes, 2012), as well as the mature and terminal differentiation of Schwann cells and oligodendrocytes (Glasgow et al., 2014; Reiprich et al., 2017). SOX10 also participates in the regulation of embryonic development and cell fate, and its expression and functional abnormalities are closely related to the apoptosis, migration, proliferation, invasion, and metastasis of various tumors, suggesting that SOX10 may play a principal role in tumor development biology. Consequently, SOX10 can be used as a potential target for cancer treatment, which is helpful for oncologists and pathologists to seek new developments for the diagnosis and therapeutic strategies of tumors. This article is devoted to the theranostic roles of SOX10 and may facilitate future research and progress in therapeutic oncology.

## Author Contributions

CD and SJ designed the manuscript. LY and FP drafted the manuscript. YC designed and drawn the figures. DS, FP, and SJ discussed and revised the manuscript. All authors read and approved the final manuscript.

## Conflict of Interest

The authors declare that the research was conducted in the absence of any commercial or financial relationships that could be construed as a potential conflict of interest.

## Abbreviations

SOX10: sex-determining region Y-related high mobility group-box 10
NC: neural crest
OLIG1: oligodendrocyte transcription factor 1
EGR2: early growth response protein 2
Ets1: ETS protooncogene 1
MCS4: multiple-species conserved sequence 4
PAX3: paired box gene 3
MITF: microphthalmia-associated transcription factor
ERBB3: epidermal growth factor receptor 3
Dct: dopachrome tautomerase
MBP: myelin basic protein
BMP2: bone morphogenetic protein 2
GGFII: glial growth factor II
NRG-1: neuregulin-1
FGF: fibroblast growth factor
hNCCs: human ES-derived neural crest cells
Tyrp1: tyr-related protein 1
TGF: transforming growth factor
MAPK: mitogen-activated protein kinase
ERK: extracellular signal-regulated kinase
MEK: phosphorylating kinase
MIA: melanoma inhibitory activity
MCM5: minichromosome maintenance protein 5
Fbxw7 α: F-box and beta-transducin repeat domain containing 7 α
CDK: cyclin-dependent kinases
shRNA: short hairpin RNA
E2F1: E2F transcription factor 1
TNBC: triple-negative breast cancer
EMT: epithelial mesenchymal transition
CD133: prominin-1
HCC: hepatocellular carcinoma
TCF4: transcription factor 4
siRNAs: small interfering RNAs
NPC: nasopharyngeal carcinoma
qRT-PCR: quantitative real-time PCR
HMB45: human melanoma black 45
EGFR: epidermal growth factor receptor
Dim: dimerization domain
HMG: high-mobility group
TA: transactivation domain
K2: K2 domain.
